# Security of medical images for telemedicine: a systematic review

**DOI:** 10.1007/s11042-022-11956-7

**Published:** 2022-03-22

**Authors:** Mahmoud Magdy, Khalid M. Hosny, Neveen I. Ghali, Said Ghoniemy

**Affiliations:** 1 Department of Digital Media Technology, Future University in Egypt (FUE), New Cairo, Egypt; 2 Department of Information Technology, Zagazig University, Zagazig, 44519 Egypt; 3 Department of Computer systems, Faculty of Computer and Information Sciences, Ain Shams University, Cairo, Egypt

**Keywords:** Medical images, Watermarking, Steganography, Cryptography, Attacks, Copyright

## Abstract

Recently, there has been a rapid growth in the utilization of medical images in telemedicine applications. The authors in this paper presented a detailed discussion of different types of medical images and the attacks that may affect medical image transmission. This survey paper summarizes existing medical data security approaches and the different challenges associated with them. An in-depth overview of security techniques, such as cryptography, steganography, and watermarking are introduced with a full survey of recent research. The objective of the paper is to summarize and assess the different algorithms of each approach based on different parameters such as PSNR, MSE, BER, and NC.

## Introduction

Medical imaging like Magnetic Resonance Imaging (MRI), Computed Tomography (CT), X-rays, and ultrasound. Has an essential role in diagnosing a wide range of diseases. Rapid improvements have been made on the internet for sharing and transferring massive amounts of information. Especially for telemedicine services development like telesurgery, teleconsultation [43], and badly need for exchanging medical images between patients’ doctors and scan centers. These medical data should be transmitted in a secure communication medium to protect patients’ sensitive information during transmission of medical images; if the transmitted medical image [87] is captured and tempered [150] by the attacker will lead to a false diagnosis issue [145]. So, confidentiality and integrity [54] became a significant challenge in medical image transmission. So, more attention is needed to secure the medical images transferred over a public network. Standard techniques used in medical image security are cryptography, steganography, and watermarking.

**Fig. 1:**
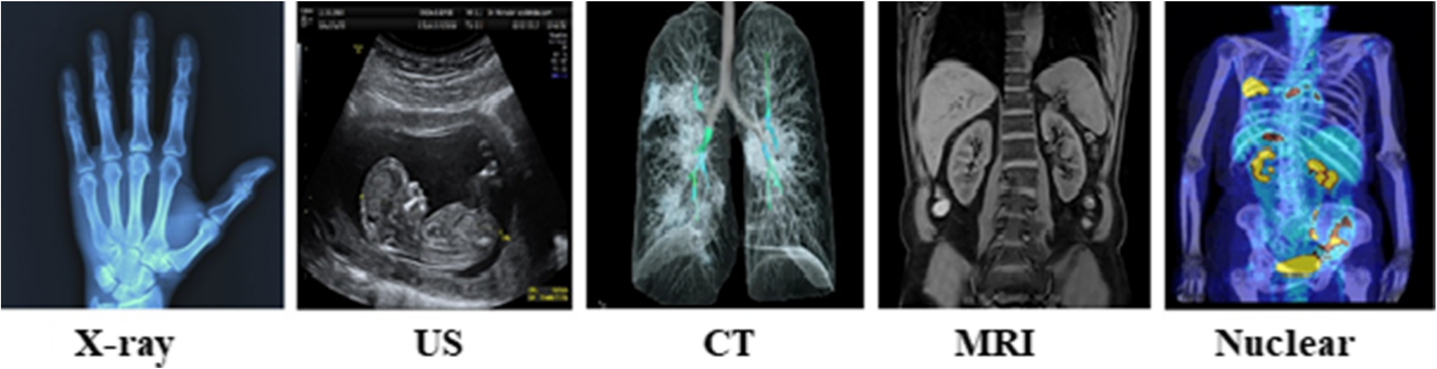
Different Medical Imaging Modalities

As of late, every year, medical companies acknowledge extra medical imaging machinery and propose advanced devices and contemporary technologies that have become a revolution. Today, a broad scope of medical imaging technologies in the world of diagnosis, as listed in Fig. 1 helps physicians obtain high-quality images for more accurate disease diagnosis.

This medical information can be downloaded with no approval from the owner. These assets cause several different problems. For example, security, proof of ownership, and copyright protection. Sensitive images carry extensive important information and different features compared to standard images. Medical images have much more sensitive and essential information than any other digital image. Each pixel in the image can be necessary for the diagnosis process, and any deformation can result in a faulty diagnosis [44]. The most robust securing of these images affects an image to the extent that it can be ignored; this is different from insensitive imagery as the border of redundancy is very low. The embedding capacity in medical images is deficient. Because medical images have a specific nature, they consist of two sections called the region of interest (ROI) [139, 195] and the region of no interest (RONI). ROI is considered an important area as vulnerable and sensitive information is present, so pixels values are critical in the diagnosis process. Because of this, the ROI is not allowed to be modified. On the other hand, RONI, or more specifically the image’s background, is the region that does not contain vital information [194].

Computer-aided diagnosis (CAD) becomes a powerful standard digital image processing tool for identifying common diseases from medical images as cancers. Role in this research area. In this research [117], the authors proposed an automatic system for identifying acute lymphoblastic leukemia by applying two steps to the medical images segmentation and feature extraction and classification. The first step is based on segmenting the white blood cells (WBCs). The second one extracts some essential features from the segmented part as statistical, shape, and geometrical. The extracted features are classified by applying various techniques to detect normal and abnormal cells. After applying the algorithm to the IDB dataset of microscopic images of blood, the experimental results demonstrate that the proposed algorithm classification accuracy rate = 97.45%.

Multi-label classification of X-ray images. Karar et al. [86] proposed a new CAD framework based on deep learning classifiers to enhance the diagnosis performance of suspected COVID-19 diseases from X-ray images. The authors utilized eleven convolutional neural networks, as Residual Neural Network (ResNet) and Visual Geometry Group Network (VGG). The experiment showed that ResNet50V2, Dense Neural Network (DenseNet169), and VGG16 models achieved the best detection accuracy of COVID-19. Hizukuri et al. [65] developed a computer-aided diagnosis (CAD) scheme on MRI (DCE-MRI) dataset by using a deep convolutional neural network (DCNN) with Bayesian optimization. The classification accuracy results of the proposed DCNN model are more significant than other conventional models that achieve high classification performance in diagnoses of masses in breast DCE-MRI images.

Forensic is considered one of the fields that need image security, not just securing images, identifying the existence of image modifications, and determining the manipulation operation applied to this data. This achievement is still a challenging problem. This paper [101] uses a convolutional neural network (CNN) model for detecting image operators in the forensics framework. Two CNN architecture is utilized to detect local noise evidence and tamper artifact evidence. The authors applied their framework to JPEG compressed images. The experimental results show that the proposed model has high detection accuracy and, in some cases, can determine the order of manipulation that previous works can’t identify.

The term “biometrics” is derived from the Greek words “bio” (life) and “metrics” (to measure). Biometrics has been traced back thousands of years; one of the oldest examples is the human face. Biometric systems identify individuals based on unique features, such as fingerprints, voice, iris, face. Deep learning has a significant role in the security of biometric systems.

Biometric systems have become widely used across a large application scale for identity verification and payment authorization. Also plays an essential role in the medical image security field. A brief introduction of some security techniques that use biometrics is discussed below.

Shen et al. [157] proposed an encryption method passed on the human face biometric by generating the chaotic face phase mask (CFaPM). The medical image ROI has been encrypted using C-Means Clustering (FRFCM).

**Fig. 2:**
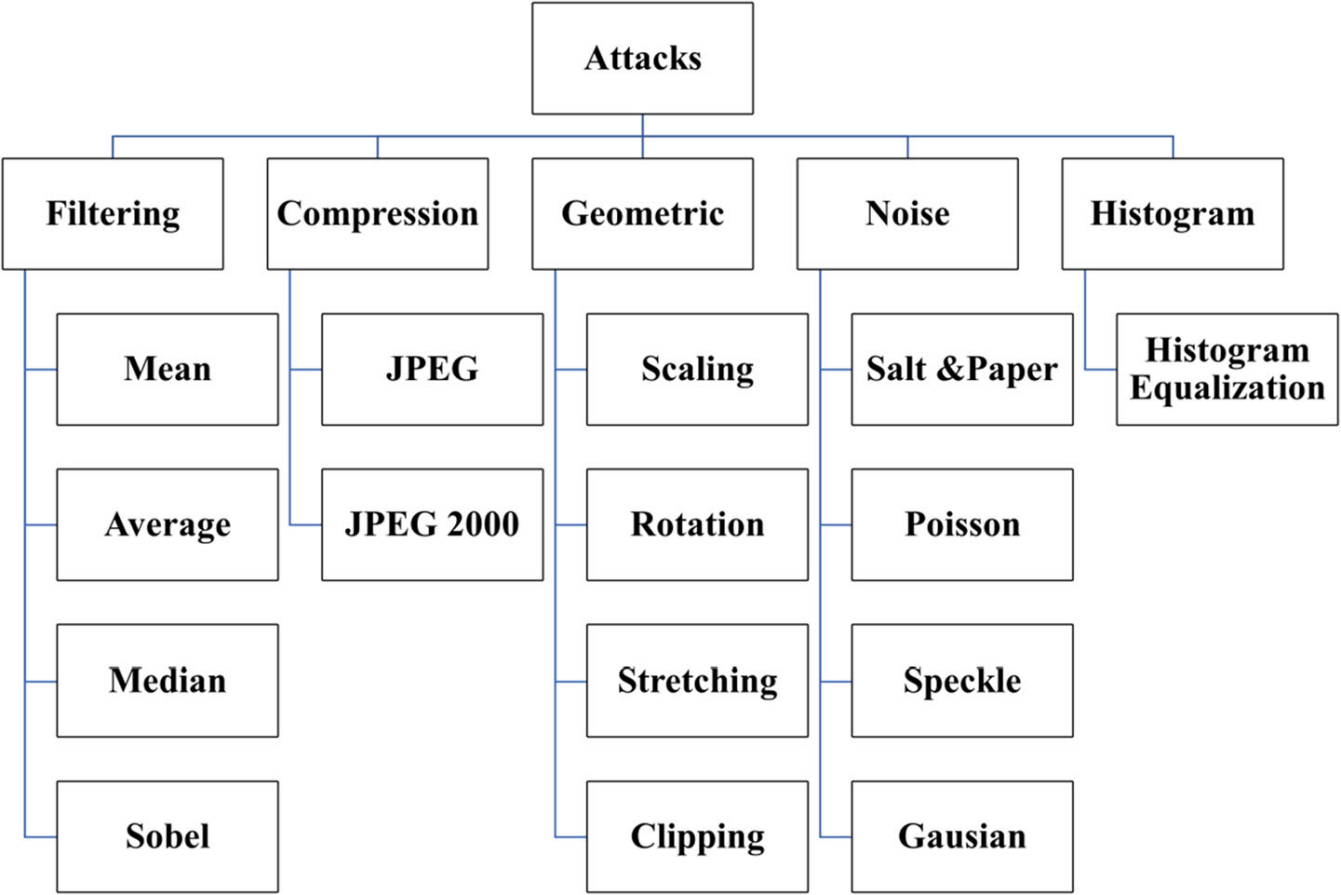
Attacks

Shams et al. [180] proposed a real-time, invisible, and secured protection method based on biometric fingerprint, further used in the recognition process for allegation ownership. This technique achieved a higher level of robustness against various attacks. Dalila Cherifi et al. [8] proved that brain MRI could be used as Security Biometrics (Hidden Biometrics), which is considered as a very robust anti-spoofing form, based on the extracted features from the brain MR images form what is called “Brainprint,” or “Brain code,” which can be used as a signature for recognition purposes. As shown in Fig. 2, many kinds of attacks can affect medical images during transmission via eHealth networks. The definition of Attacks on secured images is different from normal attacks on any transmitted data. Attacks here are not to get information from ciphers but to pollute data and deform the secured media. These are classified into geometric attacks [132] like scaling, rotation, translation, cropping, and stretching. Signal processing attacks [104, 165] as histogram equalization (HE) [192], contrast adjustment (CA), gamma correction (GA), and adaptive histogram. On the other side, image filtering [200], such as median, average, and Sobel filters, are examples of denoising attacks. Additionally, image compression techniques are other kinds of signal processing attacks as JPEG compression. Some of these attacks are shown in Table 1.

**Table 1:**
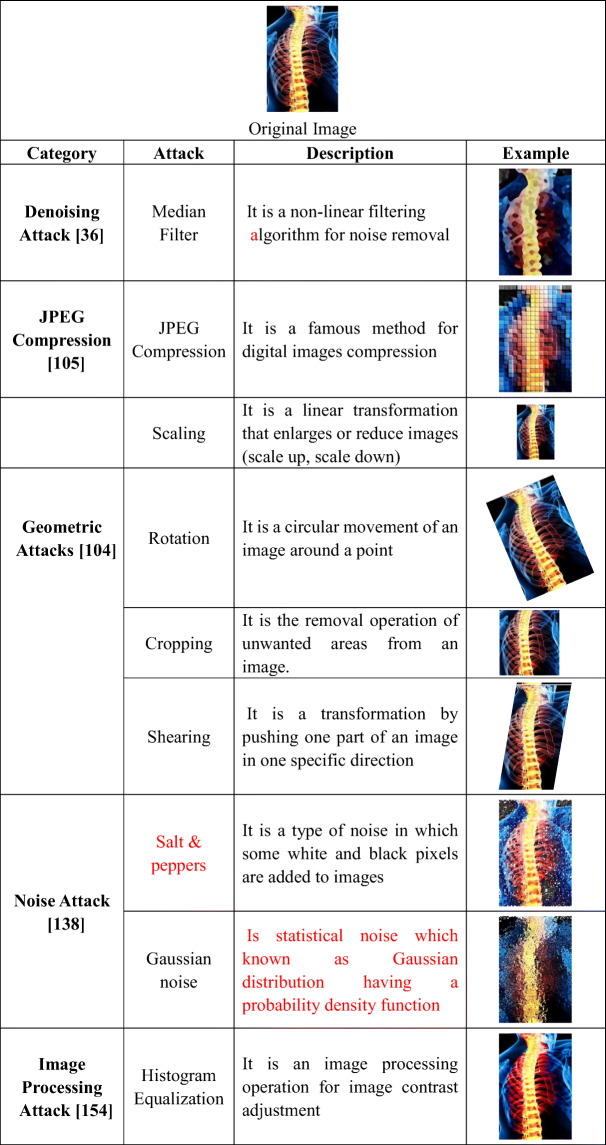
Image attacks

**Fig. 3:**
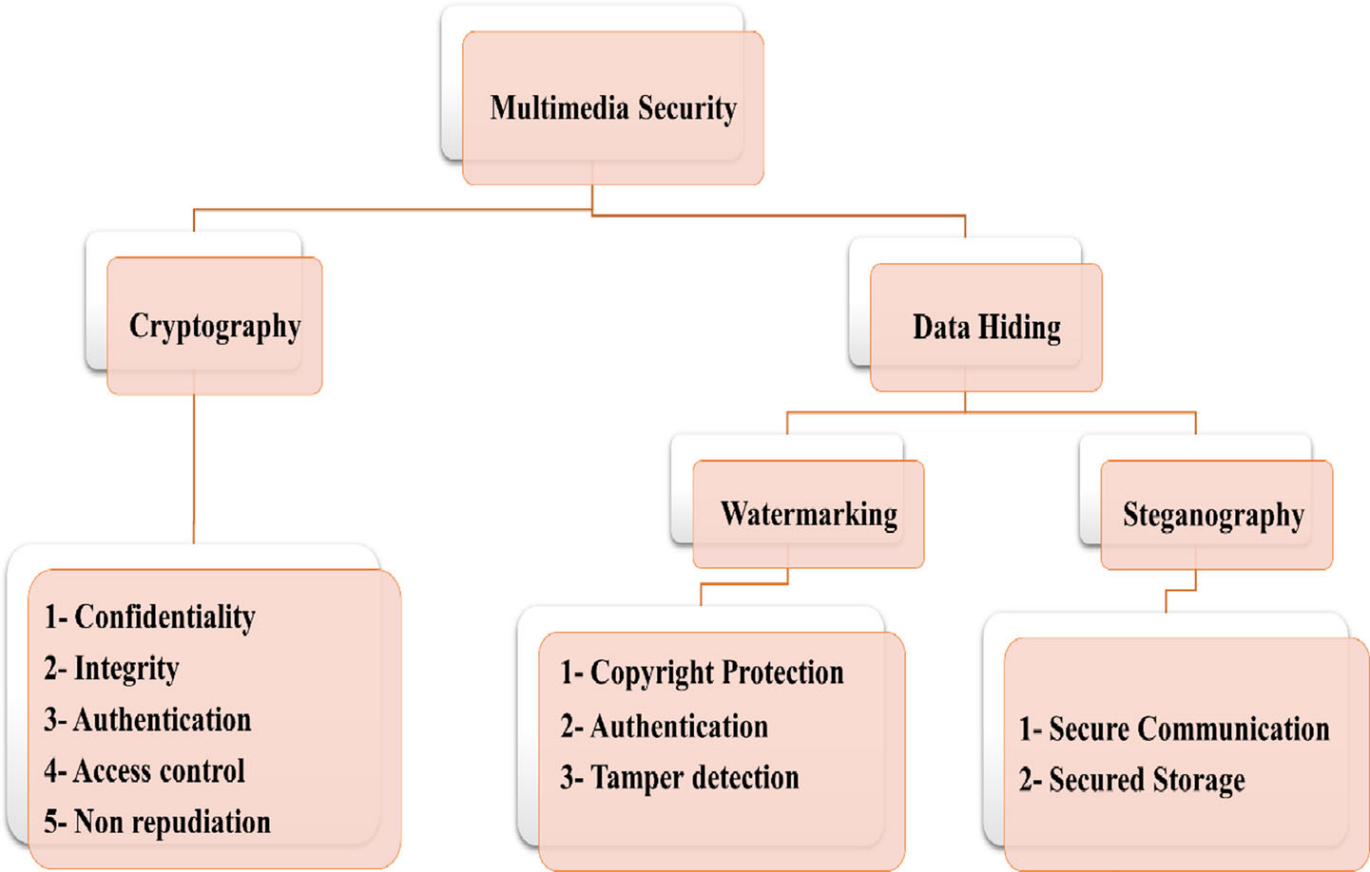
Multimedia Security approaches

Researchers present different data security techniques as cryptography and data hiding to guarantee data verification [171], as shown in Fig. 3. The data hiding [93] approach is classified into watermarking and steganography to load additional data into image pixels. In most of these techniques [99], the reconstruction phase’s container image cannot be reconstructed due to the losses during the embedding or compression process.

Medical image security has become an essential requirement for eHealth applications, including storage, retrieval, identity theft, and data management. For this reason, a lot of research work and surveys have been published to discuss medical data security approaches and copyright protection. These surveys focused only on one or two approaches, such as cryptography [37, 171], steganography [44, ], and watermarking [114, 162]. The surveys do not cover all approaches. This paper presents a study on these approaches, properties, techniques, and evaluation criteria.

The remainder of this paper is organized as follows: in section 2, we present recent cryptography techniques used in medical data security. In contrast, various techniques and types of steganography are discussed in section 3. Section 4 provides a detailed survey of recent image watermarking techniques, while performance measurement metrics and datasets are presented in sections 5 and 6. Finally, sections 7 and 8 discuss and conclude this paper.

## Cryptography

**Fig. 4:**
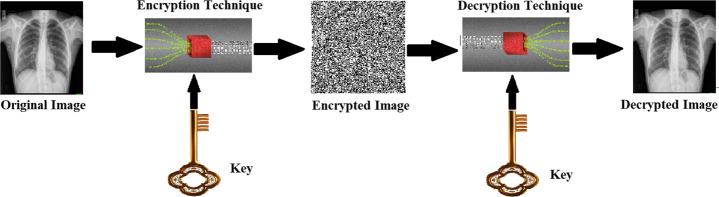
General Cryptography (Encryption and Decryption) Scheme

Cryptography [191] targets secure the communication channel. It provides data encryption techniques so that only the person who has the decryption key can decode the encrypted message. It benefits in preventing any changes or updates by an assailant in the communication medium. It is accomplished using hash functions and a public-key cipher. Most cryptographic techniques, such as Blowfish, DES, AES, and RSA guarantee a maximum level of privacy. The hybridization of these methods is also used to accomplish maximum security. While these techniques are essential to encrypting text data, they are inefficient for image security since images have essential features, such as numerous redundancies and a potent correlation between neighboring pixels. Hence, images need an effective method to attain robust security [10]. Cryptography [41] typically passes through two stages known as encryption and decryption, as presented below in Fig. 4. Encryption is a method to convert data to incomprehensible format by unlawful users using cryptographic algorithms to protect the data. The original image is transformed or encoded into an unreadable format using a secret key in the encryption stage. In decryption, the encoded image is decoded into the original image using the same key. The encrypted data is then transmitted through an unsecured channel towards the destination.

Stream cipher and block cipher are modes of encryption. When the data encoded is limited, stream ciphers are used. When the data size is large, they are chunked into blocks, and then encryption algorithms are applied, as presented below.

Different encryption techniques are proposed. Image encryption techniques can be categorized into the position-substitution-based algorithm, value transformation, and position permutation-based algorithm [37] or classified according to domain spatial and transform. In this survey, we are focused on domain classification, as shown below.

Therefore, medical image security is an essential responsibility. We need to achieve information or network security goals, such as confidentiality, integrity, and availability (CIA). Confidentiality refers to information that is secure and not accessible to an unlawful person. Integrity means the accuracy of data. Availability refers to the information being accessible to the authorized personnel.

**Fig. 5:**
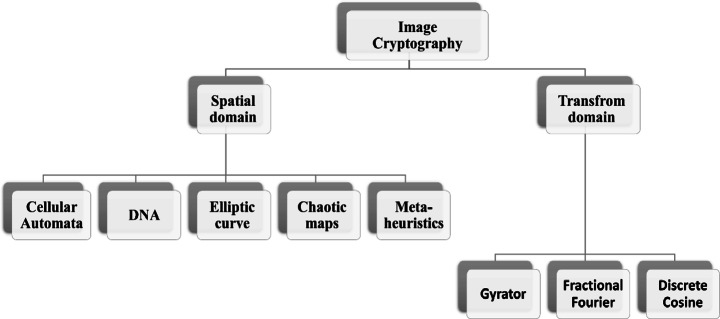
Image Cryptography Domains

Network security is not acceptable for reliable information communication like audio, text, video, and digital images. The multimedia security scope is categorized into two parts, cryptography and information hiding algorithms. The information hiding methodologies are also classified into steganography and watermarking. As shown in Fig. 5, a large number of researches about medical image security have been proposed. A brief introduction of some cryptography techniques is discussed below.

### Cryptography in spatial domain

#### Chaotic-based medical image security methodologies

Chaotic maps should be considered in the effective medium as they display a chaotic manner. This process means that any slight modification in primary conditions can generate violent change in outputs. Chaotic maps are widely used in security. The chaotic-based encryption method has many pros, such as sensibility to initial conditions, ergodicity, and necessity [88]. Therefore, chaotic maps are used on a large scale in different implementations. Some of the chaos-based encryption methods have been illustrated below.

Rajendran et al. [135] proposed a chaotic-based cryptographic architecture for medical images security during storage and transmission. At first, the key is generated by applying the chaotic map technique to the medical image. Secondly, both row-by-row and column-by-column confusion is executed. Furthermore, the diffusion process is performed using binary reverse and complement operation. Diffused images and chaotic key images are XORed. The proposed system security level is measured by applying different attacks. Simulation results indicate that the developed cryptosystem has to satisfy the requirements of IoT healthcare applications. Harshitha et al. [64] proposed an image encryption technique for medical images based on a chaotic logistic map and linear feedback shift register to generate pseudo-random sequences, then XORing these sequences to construct the key used for encryption generate cipher medical image data. The proposed scheme is robust to various attacks and secures many formats of medical images.

Pak and Huang [128] proposed a technique that cures the issue in a single chaotic map. In [84], the authors proposed a new image encryption algorithm for both gray and colored medical images, wherein the proposed algorithm surpassed other existing encryption methods.

#### Deoxyribonucleic acid (DNA)-based medical image encryption techniques

DNA technology began receiving significantly more attention a few years ago. Affected various fields, such as information science, medical systems, etc. DNA consists of a genetic code in which data is stored and converted to various genetic codes. Recently, a simulation environment has been designed based on DNA technology for biological experiments. This concept has been advanced in the expansion of DNA in the encryption field [197]. Massive storage, enormous parallelism, and low power exhaustion are the significant reasons for using DNA in the encryption of images [187]. Some DNA-based techniques are demonstrated in the following discussion.

Banu et al. [29] recommended a new medical image encryption technique for DICOM images based on Integer wavelet transform (IWT) and (DNA) sequences. Logistic map utilized for random keys generation. The results confirm that it is robust against various attacks. Mishra et al. [116] introduced a medical image security system using bit-level diffusion and DNA coding, also pseudo-randomly based on Logistic Sine utilized for key generation to make the secured image more robust. The experimentation proved that the original image could be recovered without any data distortion.

Guesmi et al. [58] introduced a hybrid cryptography model of hash algorithm SHA-2, chaotic map, and DNA masking for medical image security. Since the information entropy is an essential feature of randomness, the proposed approach works on improving entropy. So the theoretical analysis showed that the algorithm is robust against typical, statistical, and exhaustive attacks.

To overcome digital medical image threats, Ravichandran et al. [137] address an encryption technique to medical image security based on DNA hybrid with chaos and IWT. The technique consists of two stages diffusion and shuffling phases. The row and column of the image are shuffled circularly bitwise shifted to enhance the security against attacks, then the diffusion stage start based on DNA XOR. The proposed model was tested on 100 DICOM medical images to evaluate it against differential and statistical attacks, which showed that it overcame other techniques.

This paper [16] proposes a secure medical image encryption scheme based on DNA), chaotic maps, and hash functions (SHA-256 and MD5). The algorithm is applied in three stages: key generation, second rotation, and permutation, third DNA encoding-decoding. In the key generation stage, the key is generated based on original image metadata. Rotation and permutation applied on two MSB bits of the medical imageTo remove the black background. The third part is the DNA encoding-decoding utilized through the logistic map. Security analysis of the proposed scheme proved that the scheme is robust against common attacks. Furthermore, a large key space.

El-Shafai et al. [47] present efficient cryptosystem-based deoxyribonucleic acid (DNA) in addition to logistic chaos maps and piecewise linear chaotic maps. First, the secret key is generated using PWLCM. Then, the input image is encoded with the logistic chaos map, and the secret key image is encoded using DNA rules. Finally, these steps are iterated on image columns once again to get the best-ciphered image. The experimental results showed that the model has high security with low time complexity. Furthermore, it can stand against different types of attacks.

#### Elliptic curve based techniques

Yin et al. [196] proposed a medical image cryptosystem that utilizes an elliptic curve with homomorphic encryption. The authors in [153] discussed a new enhanced cryptographic scheme for medical image protection generated from IoHT healthcare devices using an advanced optimization technique, based on elliptical curves and Grasshopper Particle Swarm Optimization (GOPSO) in selecting the optimal key to enhancing medical Image security. The theoretical and practical results proved that the algorithm is secure and robust against various attacks compared with other optimized encryption algorithms. The experimental results show higher key sensitivity, better encryption accuracy, and high resistivity for statistical attacks.

This paper [32] addresses a new cryptographic technique for medical image protection based on combining ECC with Hill cipher (ECCHC). The authors do this combination to overcome the weakness of some encryption schemes against some attacks and the key length challenge that is not robust against brute force attacks. The efficiency of the proposed is evaluated through security tools and exhibits better security features than the state-of-the-art techniques.

Koblitz and Miller [121] designed a key-encryption algorithm based on the elliptic curve. The better complexity and small key size are the main aspects of the elliptic curve encryption technique because the elliptic curves technique is based on algebraic curves’ features.

#### Metaheuristics-based techniques

Metaheuristics algorithms have an essential functionality in NP-Hard problems optimization. The importance of this algorithm lies in the constant parameter optimization used in encryption cryptography. EA’s ability to evolve multi-appropriate outputs in single development is based on population manner [97]. However, this technique also has weak points, such as low convergence speed, etc.

In this paper [184], a secured algorithm for medical Image security. A medical image is considered a watermark and encrypted using encryption algorithms. The encryption is done using AES and RSA encryption algorithms. Different metaheuristic approaches like Genetic Algorithm (GA), Bacterial Foraging Optimization (BFOA), and Differential Evolution (DE) are proposed to preserve the integrity of the medical images.

Proposed intelligent symmetric cryptography for medical image encryption based on the quantum-based key generator and chaotic map. Janani et al. [77] introduced a new quantum-based cryptosystem with two levels of security for medical images. The algorithm applied on the cancer imaging dataset, performance evaluation of the proposed algorithm ensures the medical image integrity and reinforces confidentiality. Lin et al. [106]. The algorithm was validated using a chest x-ray database, and experimental results show robustness against the passive attacks.

#### Cellular automata

Vijayakumar et al. [185] presented a cryptographic scheme based on Cellular Automata (CA) and is targeted in securing medical DICOM (Digital Imaging and Communications in Medicine) images. This architecture is implemented with a Hardware Cyclone device. The CA-based scheme consists of two diffusions and confusion. The proposed scheme is evaluated against multiple metrics, with an entropy of around 7.9975 achieved.

### Cryptography in transform domain

Image encryption methodologies based on the transform domain are considered one of the essential image encryptions. The given media is converted from spatial to transform domain using one of the transform models for the encryption process. Examples of these techniques are gyrator transform (GT), fractional Fourier transform (FrFT), and discrete cosine transform (DCT). These algorithms are discussed below in detail.

#### Gyrator transform

Yatish et al. [53] demonstrate a new cryptosystem technique based on gyrator transform and phase-truncated Fourier transform using a triplet of functions to evaluate the complex function for medical image security. The complex matrix is split into two matrices, one for the real part and the other for the imaginary part. In the next step, a random distribution function is applied by one of the triplet functions. The simulation results and attack analyses showed the effectiveness and robustness of the technique against various attacks.

#### Fractional Fourier transform

This paper [94] introduces a new scheme of medical data security based on fractional discrete cosine transform (FrDCT) coefficients with a high degree of freedom. After that, a chaotic map is applied to FrDCT coefficients. Experiments applied to study the efficiency of the proposed method after that have been compared with state-of-the-art techniques showed that surpass is more efficient than other algorithms.

#### Discrete cosine transform

The authors in [7] proposed a chaotic-based encryption technique that combined discrete cosine transform (DCT) for medical images. The medical image is compressed twice, one by using DCT. And again using the arithmetic encoding technique. After that, the compressed image is encrypted using a chaotic sequence to scramble the output. The experimental results showed the efficiency of the proposed model with a PSNR value equal to 41.70 dB.

Fang et al. [51] illustrated a new encryption algorithm to secure the host medical image to protect private information; the author’s algorithm is based on Bandelet Transform and Discrete Cosine Transform. The medical image is first encrypted using a Logistic chaotic map, and then the features are extracted using Bandelet Transform. The experimental results showed the robustness of the proposed algorithm and proved that it could solve the problem of information leakage.

## Steganography

Digital steganography was proposed to avoid snooping on secured transmitted data. The steganography concept was the concealment of digital data. Steganography is a Greek word consisting of two words, stegano, which means “covered” and “graphy,” which indicates “write.” So the two words combined mean “covered writing” [20]. It treats various methodologies of embedding secret data like video, image, audio, text, or files into other multimedia payloads using the private key. The concealed information is called a secret message, and the container in which the secrete file is embedded is called a cover file. The cover can be any multimedia file, such as video, audio, image, or text. The resulting file of the embedding operation is termed a stego file.

Irrespective of any efforts and enormous achievements achieved in steganography, the adoption ratio of steganography is still relatively weak in real-time applications in which privacy of sensitive information is crucial, such as with military documents, forensic reports, etc., and health records [81]. There is still a trade-off between integration capability and distortion. It is also essential to keep a minimum of recovery errors and resistance to data handling. In steganographic algorithms based on machine learning, sophisticated machine learning methodologies are utilized to equilibrium these trade-offs, even preserving high performance in the whole process [21].

**Fig. 6:**
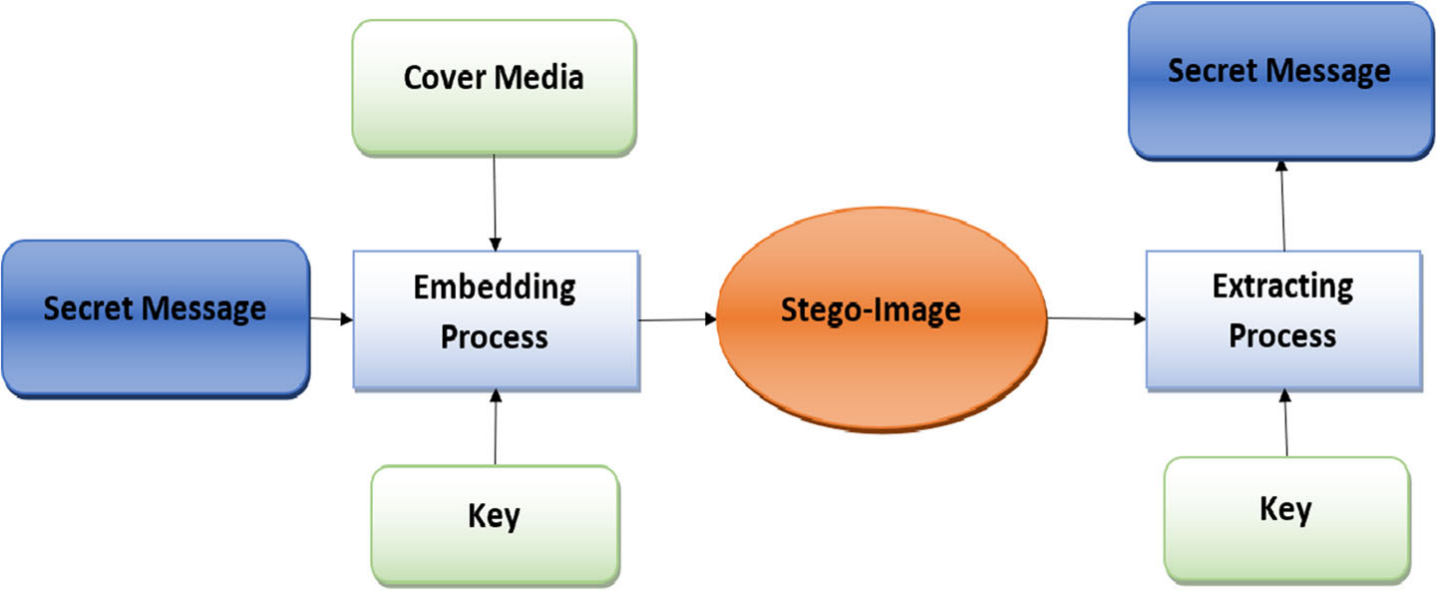
Steganography Embedding and Extraction Phase

As mentioned above in Fig. 6, steganography involves embedding an essential piece of information inside another multimedia file. Hence, steganography models need to withstand such massive information bulk, must also have a more extensive equivalent capability, called a payload, to receive the information in such a way as to maintain the image quality, called imperceptibility. A secure stego-key is used to prevent intruders and is essential to having the ability to resist any image transformations techniques that can modify or change the covered data [82]. Sathua et al. [149] mentioned that the image consists of numbers in a two-dimensional array from a computer’s point of view. These numbers represent light depth at different locations and are called pixels. The images are commonly stored in 8-bit or 24-bit format. The higher the number of bits, the higher its capacity; therefore, 24-bit images own the highest capacity for hiding sensitive data as a pixel expressed in 16,777,216 color values, as explained by Muhammad et al. [119]. Hence, image steganography uses the redundant bits in altering data without deforming the original image features. A reversible information hiding technique is proposed in [2] to preserve the visual quality of color medical images.

### Steganography system properties

**Fig. 7:**
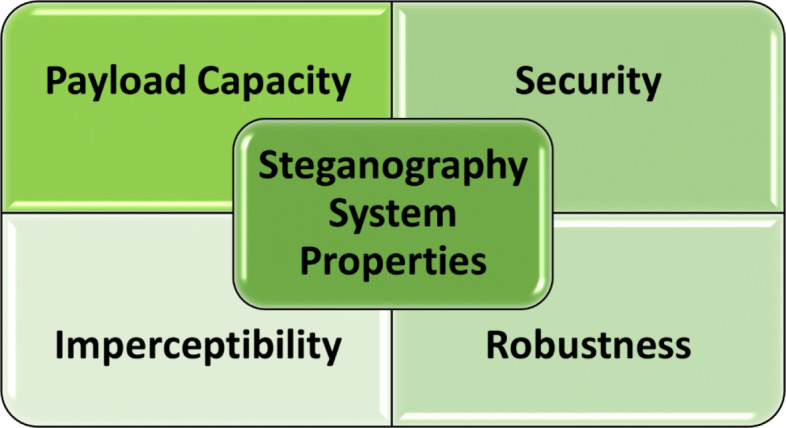
Steganography System Main Properties

The authors of [22, 189] show three fundamental properties for a successful steganographic system. These properties are security, imperceptibility, and cover media payload capacity. There is also a fourth property mentioned in their research [13]. In addition to the properties mentioned above, robustness has been added, as shown in Fig. 7. These properties are considered the most influential criterion, so these parameters test most steganographic systems. Hence, any proposed algorithm should maintain most of these properties. Although the robustness property is not required in many applications, the other three properties are typically required [183].

#### Imperceptibility

Steganographic methodologies’ strength resides in hiding secret information in digital data, so naked eyes nor statistical analysis can detect it. But also, the steganographic algorithm should not affect the cover media. The methodology is secured when the statistical analysis for the covered image and the raw data are the same.

#### Security

Security is considered the main interest to protect data access while sent through the channel by attackers. In most steganographic models, the word security refers to hiding data from unidentified outsiders with no relation between senders and receivers. So, the steganography approach is considered safe if the secret data is unnoticeable by statistical analysis methodologies.

#### Payload capacity

Venkatraman showed that steganography’s primary challenge is increasing the cover media capacity without affecting the security and imperceptibility. An efficient steganographic model aims to embed max payload inside minimum cover media.

#### Robustness

In [113, 198], robustness is defined as avoiding any corruption in the stego-image and saving the embedded information’s fidelity even if the carrier image is modified by an intruder using image operations like scaling, resizing, and rotation, and others.

Payload allocation performance enhancement is still a challenge in color image steganography. In standard steganography techniques, the RGB channels are assigned equally. Still, this methodology is not accurate enough as the security performance of color images is not based on embedding algorithms. Still, payload, so Liao et al. [103] proposed a channel-dependent payload partition technique to assign the embedding capacity among RGB channels adaptively. Experimental results show that the proposed scheme effectively increases the performance of the embedding.

It is still a challenge for steganographers to balance among all these different steganography requirements. The quality of the stego-image and the payload capacity of the cover image are at odds with each other, as it is challenging to maintain the imperceptibility and increase capacity simultaneously [4]. The requirements are affected by changing the cover image pixels after embedding process secrets. In recently proposed steganography techniques, slight changing of the cover image after embedding while preserving payload capacity, which indicates a high steganography algorithm, has been used to indicate higher stego-image quality and lower message detectability. The embedding efficiency indicates the ratio of changed cover images pixels by calculating the number of embedded bits per embedding change. Robustness against steganalysis increases When the embedding efficiency increases and is less detectable.

### Steganography approaches

**Fig. 8:**
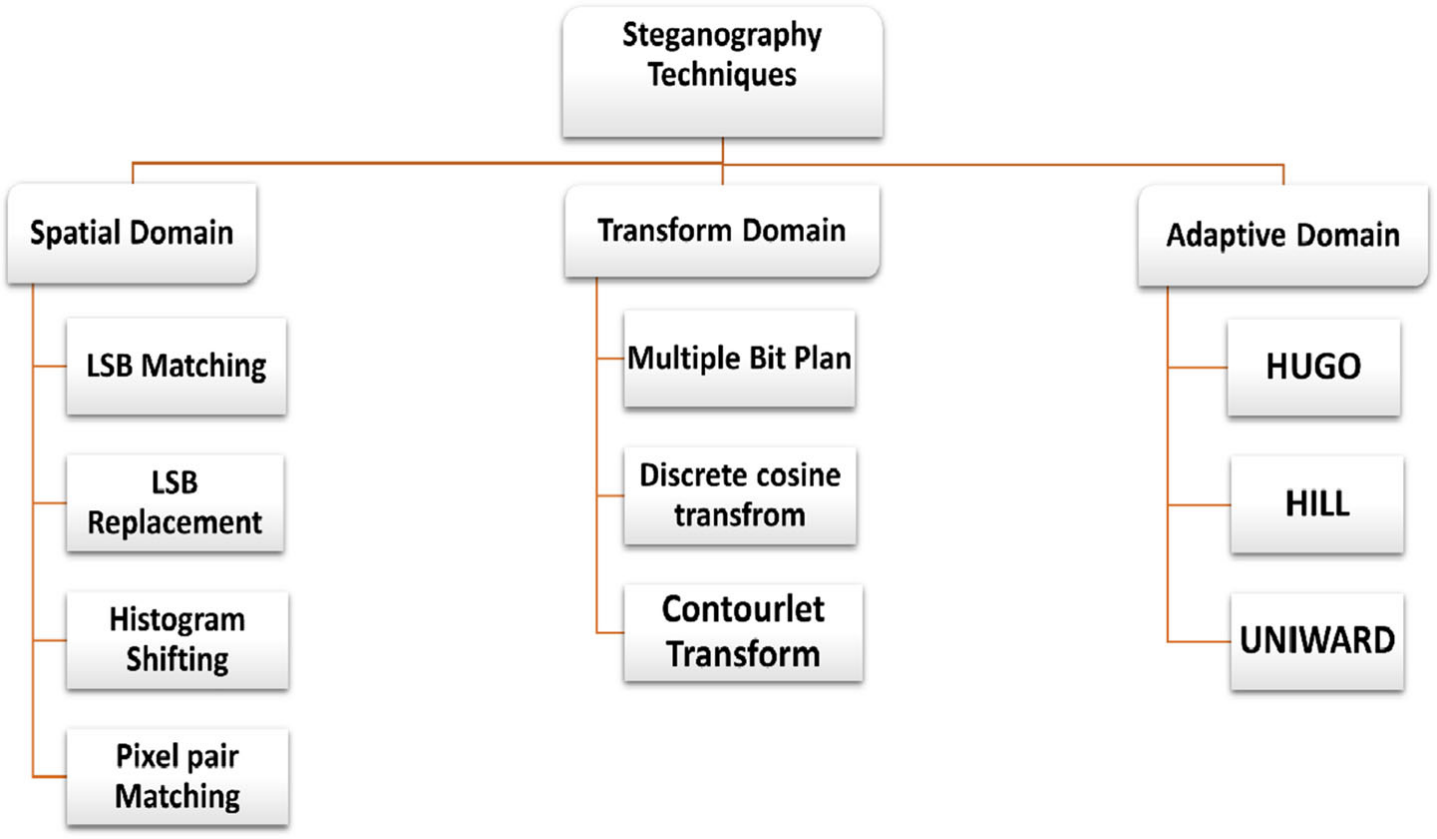
Steganography Working Domains

The primary characteristic of steganographic techniques is stego-image security while transmitting on the channel. Various techniques for steganography were utilized based on the nature of the embedding process. Hence, steganographic techniques can be categorized into spatial, transform or frequency, and adaptive domains, as presented in Fig. 8. In the following sections, this later classification will be discussed more in-depth with some recent research [96].

Abdulla et al. [6] proposed a two-step steganography mechanism to enhance undetectability of hidden data and quality of stego-image, first image size is reduced using SISR algorithm and second step embedding using bit-plane mapping algorithm based on the Fibonacci representation intensities, the experimental results proved that the new algorithm increased the robustness, increase capacity, and reduce image degradation.

#### Spatial domain steganography

This domain’s techniques are considered the most straightforward and convenient method of information embedding in digital images by altering the carrier image’s pixel rate to insert the secret information bits. These methodologies have little complexity of extraction and embedding. Some of the primary spatial domain methodologies are discussed below. Some examples from research papers are explained [82]. From these multiple-bit planes based examples, Collines et al. [42] demonstrate a method that uses pixel bit-plane values for sensitive information. Slicing the system into multi-bit planes using the ANR255 sequence is the first step in the proposed technique.

The information has been encrypted before embedding to achieve more security. After the embedding stage, the cover media is turned into an 8-bit representation to be used as the stego-image. This technique has more than one advantage. The first is the embedding capacity. Another advantage, the randomness of embedding is very high and provides robustness for the system against the steganalysis operation. The main loophole lies in the vulnerability that the stego-image is susceptible to contra-attacks, and any slight alteration in pixel value position may deform the embedded information.

The authors in [5] proposed a hybrid technique based on two well-known schemes, LSB randomly and 2LSB, to increase the payload capacity, un-detectability, and stego quality. Experimental results proved the robustness against most statistical attacks as MLSB-WS, WS, and PoV.

To overcome the existing Least Significant Bit (LSB) steganography. The effective reversible data hiding technique and histogram shifting are used in the proposed technique [17] for hiding data in encrypted bio-medical images. Histogram expansion and histogram shifting have. The Paillier cryptosystem algorithm encrypts each pixel. The testing results showed that the embedding rate in the proposed model is higher than the existing scheme.

Santoso et al. [148] proposed a new steganography technique using Pixel Value Differencing (PVD) algorithm for hiding sensitive data in medical images. Based on the experimental results of testing, it is found that the proposed algorithm-produced images have better PSNR value than others implemented using LSB.

Stoyanov et al. [168] introduced a stego hiding scheme for medical images based on the nuclear spin generator, the efficiency of output images using the proposed algorithm measured using MSE and PSNR metrics, and the results show that the new steganographic scheme gives good performance with medical images security.

The embedding process can change the medical image structure to overcome this effect. The authors in [151] proposed a reversible data hiding technique based on shifting histograms and RC4. The experimental results get high SSIM and PSNR, which means that the medical image can be retrieved successfully.

This paper [62] presents a new hybrid steganographic method based on Lempel Ziv Welch (LZW) and AES encryption for medical image security. Firstly the secret information is compressed using LZW and then encrypted using AES. In CEDE, after that, the encrypted image is embedded. The proposed technique provides high security with a high capacity compared with existing methods.

#### Transform domain steganography

The transform or frequency domain decomposes the media file into frequency coefficients before embedding essential data. This technique has many advantages for robustness against attacks. It can resist such types of attacks that can alter hidden data and imperceptibility and the capability to withstand distortion in the stego image [20]. In addition, the frequency domain also has cons, such as a lower payload than the spatial domain with high computing complexity, as Kaur and Pandey [89] mentioned in their research. In transform domain-based steganography, Fourier information has been used during the reconstruction process, which is considered a flawed tool in reconstructing the signal. Some of the used methods include integer wavelet transform (IWT), discrete cosine transforms (DCT), discrete wavelet transforms (DWT), and discrete Fourier transform (DFT).

Discrete Fourier transform is widely utilized in different image processing algorithms. DFT is a common transform technique used in signal processing. In DFT, a signal is decomposed into sines and cosines components. In image steganography, these components can be modified to be an essential tool for data hiding. Modified coefficients are returned to the original image and considered as the stego-image.

The received image is analyzed again into its transform components, and covered information is extracted from the recipient. Different steganography-based systems have been mentioned [82].

The authors in [143] proposed an LSB-based steganography technique for medical images with an encrypted DNA algorithm. The encryption algorithm applied on the patient data in the spatial domain, the proposed algorithm applied on chest x-ray images of the COVID-19 patients. From experimental results, it is evident that the algorithm shows greater robustness than others.

The authors in [131] proposed two-layer security using the LSB steganography technique with encryption for medical images. The experimental results showed that the proposed technique could preserve integrity and confidentiality compared with other hiding techniques with one level of security.

Reshma et al. [140] proposed an efficient steganography scheme for medical image security using support vector neural network (SVNN) and DWT to overcome the complex issues found in existing techniques as LSB. The SVNN classifier is utilized for selecting suitable pixels for embedding. The experimentation showed that the proposed model achieved high performance.

Thanki et al. [177] proposed a hybrid technique between steganography and cryptography for medical images in this paper. The embedding process is implemented using singular value decomposition (SVD) and discrete cosine transform (DCT) in this approach. The testing operation was done under various image processing attacks, and the experimental results show that the proposed algorithm has low computation time with higher imperceptibility of other existing approaches.

In this study, Arunkumar et al. [18]. Proposed a robust hybrid steganographic approach using the chaotic map, Singular Value Decomposition (SVD), Redundant Integer Wavelet Transform, and Discrete Wavelet Transforms (DCT). SVD and DCT achieve a higher level of imperceptibility and extra security and robustness achieved by chaotic map. By comparing the introduced method, the results applied on the UCID database show the effectiveness of this algorithm in geometric transformation resistance compared with other existing.

Jeevitha et al. [78] proposed a steganography approach for medical images based on Hidden Markov Tree Contourlet transform, canny edge detection, and Paillier cryptosystem. Firstly the cover image is mapped to the frequency domain using HMT and then applied to detect smooth image edges. The embedded information is encrypted with a pallier cryptosystem, and the Particle Swarm Optimization is utilized to select the embedding location. The experimental results show that the proposed method provides robustness compared with other techniques.

#### Adaptive domain image steganography

Subhedar & Mankar [169] mentioned in their research another name for the adaptive domain steganography, which is “statistics-aware embedding” and is also called “Model-Based” [146]. An adaptive method is an entire case of transforms and spatial methods. This method can select random adaptive pixels based on the cover image and select pixels with a more considerable standard deviation value for each block individually. Hajduk and Levický [60] also demonstrated that a global statistical characteristic was evaluated before interacting with the image coefficients. This statistical coefficient’s importance is for finding areas in the cover image that can be altered without image deformation. Hence, being able to neglect smooth areas that have similar coloring. This process means that images with a combination of colors are excellent for adaptive steganography techniques. Liao et al. [102] proposed two payload allocation methodologies based on distortion distribution (ES-DD) and image texture complexity (ES-ITC) to investigate payload distribution in images steganography. Experimental results proved that the proposed methodologies could perform better in multiple image steganography but not on JPEG images.

According to the mode and nature of adaptive steganography systems, they can be classified into artificial intelligence and machine learning, human visual systems, and region-based Steganography.

#### Human vision system steganography

In general, HVS is considered an approach for observing, comprehending, and processing optical information. HVS enhances the understanding of images, as the human vision has challenges in recognizing the finest details of image backgrounds. HVS has the primary advantage as it is indistinguishable, even if an intensive payload is embedded in edge regions and complex textures [100]. These techniques are mainly based on detecting the target region for hiding sensitive information using the transform or spatial domain techniques explained previously. Accordingly, researchers are working on this point to enhance the imperceptibility using various directions. Several examples of these directions are quad-tree segmentation used to detect smooth areas appropriate for covering data with minor distortions in the image [66]. The author here [109] defined just noticeable differences in the (JND) technique to precept changes while embedding process. It was observed that this category would adopt the embedding payload capacity without distorting the stego image; hence, detecting whether the image is covered or not by the intruder’s naked eye is very challenging. However, the embedding can be revealed using statistical analysis.

#### Region-based steganography

As mentioned before, the goal behind steganography techniques is to share data between the source and destination in an unnoticeable format. This way, trying to get the most uses, the most suitable regions host images for embedding secure data [25, 61]. With this technique, the embedding process focuses on edge and texture regions as the details in these regions are great [35, 39, 133]. This approach defends against image distortion due to any modifications. The main advantages of this technique include high imperceptibility and robustness, while the main disadvantage is that the embedding rate is very poor.

In this work [50], the authors proposed a hybrid security approach for medical image security. The approach comprises both Fragile Steganography and robust watermarking. The authors take care of ROI inside medical images. Firstly the features of the region of interest are computed and then embedded into the cover image. The steganalysis proved that the attacks applied on the transmitted image are not perceptible.

In [120], the high-frequency pixels used in covering secured data by the LSBM revisited algorithm has been selected according to the threshold value. In [12], the author mentioned approaches based on images’ characteristics for controlling the capacitance of hiding. The researcher in [91] used the “ant colony optimization” algorithm in cover image pixels selection, then LSB was used for the embedding process.

#### Intelligent and machine learning techniques-based steganography

Artificial intelligence (AI) and machine learning (ML) have become widely used in various advanced applications [45, 70]. Initially, it was introduced for optimization, object retrieval, and recognition purpose [190]. Later, their usage extended for other requirements like compression, classification, segmentation, enhancement, and other image processing operations [199].

It is said to be efficient for any steganographic technique if the embedding process makes a minor distortion in the stego-image with high embedding capacity, and errors in the retrieval process are kept to a minimum. Many advanced machine learning techniques are proposed to achieve this efficiency [129]. Some of the machine learning systems used are neural networks (NN) [152], support vector machine (SVM) [179], genetic algorithm (GA) [115, 182], and decision-tree-based analysis. Some of the related research works are illustrated in the following sections.

In this study [126], Fatmanur et al. proposed a steganalysis classifier approach to detect whether a given medical image contains hidden data or not. The support vector machine is used as a classifier for stego images’ pixels and cover images. As a result of the analysis, the classifier accuracy is 99.28%.

The authors in [168] illustrate a medical image steganographic approach named BOOST for medical image security, based on the nuclear spin generator. The algorithm was measured using PSNR and MSE and shows that the proposed steganographic technique can stand against different medical image attacks.

Jain et al. [73] proposed a hybrid cryptosystem and steganography approach based on a piecewise linear chaotic map and dynamic decision tree. The algorithm applied on five steps, diffusion and confusion, pseudo-random sequence using linear feedback, permutation and XORing using pseudo-random sequence, RSA encryption algorithm used for keys encryption, and dynamic decision tree for steganography algorithm. The performance analysis and histogram analysis showed that the algorithm achieved maximum embedding capacity.

With advancements in medical imaging, the need for securing this sensitive data raises. Jambhale et al. [76] proposed a neural-crypto computing steganography mechanism to achieve confidentiality and privacy of medical images. The proposed mechanism utilizes neural networks combined with RSA cryptographic algorithm. The experimental results show that the proposed algorithm improves medical image security upon existing techniques.

## Watermarking

Internet and image processing techniques have made it easier to copy, modify, and distribute digital data at a low cost and without any quality degradation. Digital image watermarking provides an alternative solution for ensuring tamper detection and ownership verification [31]. Watermarking is considered a way of embedding information in the digital content without modifying its value to recognize the original author of the data.

In this survey, a review of some recent research work in medical data security is discussed. Most recently, researchers have proposed a watermarking mechanism for the security of medical data. When medical data needs verification, watermarking can be a good solution [172]. Therefore, to guarantee originality and control disintegrate, watermarking is being used to publicize medical data security. Watermarking technique overcomes steganography’s limitations by inserting a watermark inside the cover image such that the embedded watermark cannot be discovered [176]. However, the embedding process should not affect the image’s visual quality.Digital watermarking techniques are derived from steganography, the science of communicating information while hiding its existence. The watermark is embedded in the host media so that it can’t be separated from the data. Thus, by using watermarking, the work is still available but watermarked [118].

Both watermarking and steganography belong to data hiding but have different objectives and conditions. In steganography, for example, the critical data is the internal data, and the external data is a harmless message, whereas, in watermarking, the sensitive information is the external data. The internal data are additional data for protecting the external data, which is the watermark. These methods ensure authentication, integration, tamper-resistance, and content verification of the given image [107].

**Fig. 9:**
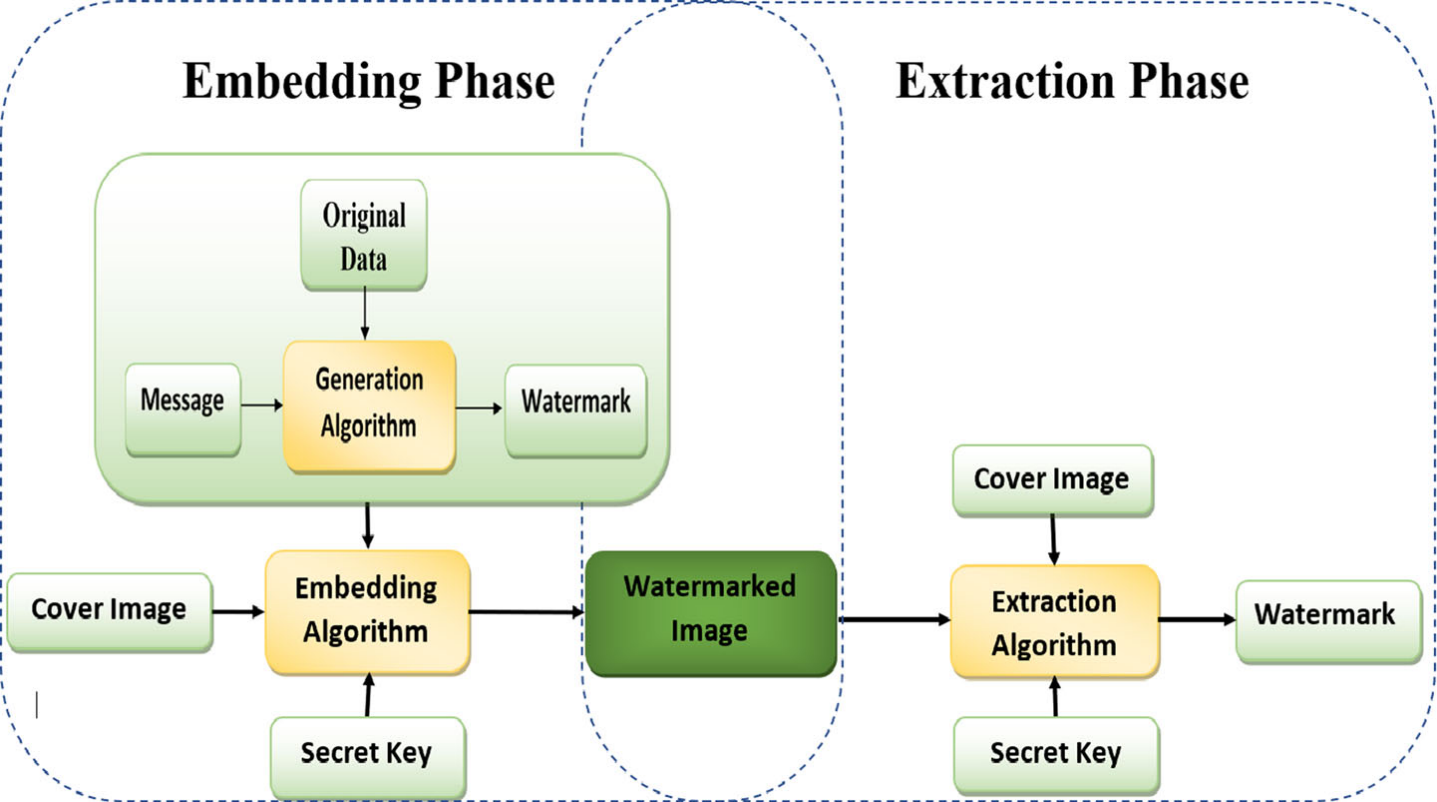
Digital Watermark System

A digital watermarking scheme consists of three phases as shown in Fig. 9: first, it generates the watermark, second is embedding the watermark, and last, extraction of the watermark. When the copyright of a digital image is doubtful of being tampered with, the owner can retrieve the image’s watermark to ensure his copyright.

### Main components of watermarking scheme

#### **Phase I:** Watermark embedding

The embedding algorithm embeds the generated watermark unique to the user into the cover image using different watermarking methodologies, such as LSB substitution, histogram shifting, etc. The watermarked image is sent to the receiver [27] to be verified.

#### **Phase II:** Watermark extraction

Watermark extraction is the process of extracting the embedded watermark from the watermarked image. The watermark image may be found to have been tampered with or not. The extracted watermark verifies the integrity and authenticity of the image and owner verification.

### Primary requirements for ordinary digital image watermarking

Robustness: This is the resistance of the algorithm against various malicious and innocent attacks. Some processing operations on the image, such as resizing, compression, and cropping, are examples of accidental attacks that affect the image [28].
Embedding capacity: Generally, in data hiding techniques, this factor is considered one of the essential requirements that indicates the size of data embedded inside the cover image without image distortion.
Fidelity and imperceptibility: Used to hide the watermarked image from eavesdroppers’ naked eye, so they cannot distinguish between watermarked and un-watermarked images. The fidelity factor indicates that the watermark is imperceptible and invisible and can be measured by SSIM or PSNR [19]. This factor should be a minimal value [170].
(IV) Computational complexity (speed): This factor is considered very important for some real-time applications. It indicates the time consumed by the algorithm in the embedding and extraction process, which determines how fast the algorithm is.

### Special requirements for medical image watermarking

In addition to the significant watermarking requirements, there are specific features for a medical watermarking system, such as:

1. **Reversibility:** This factor should be considered when dealing with medical images, as any distortion in this type of data could lead to an incorrect diagnosis. The algorithm is reversible; the recovered image is the same as the original without distortion after the embedding process.
2. **Integrity control:** This factor is used in the verification process to determine whether the eavesdropper has modified the image while being transmitted.
3. **Authentication:** This factor identifies the original author or the correct patient to whom the transmitted medical data belongs.

### Watermarking approaches

**Fig. 10:**
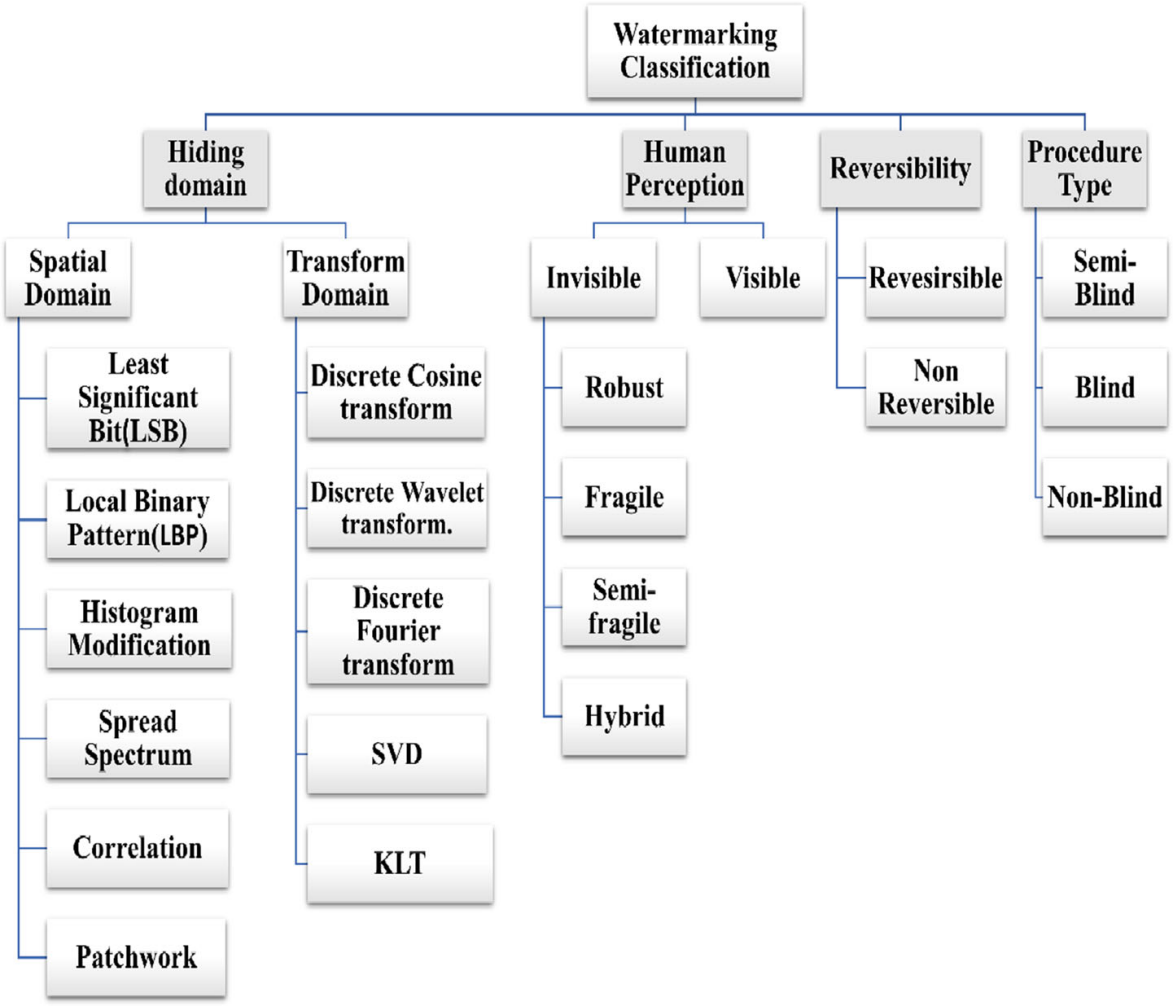
Watermarking Techniques

Watermarking techniques could be classified as shown below in Fig. 10. The watermarking classification was categorized into four categories: domain-based, human perception-based, the reversibility of watermarking technique, and the procedure type [85]. The domain-based watermarking was also classified into transform domain and spatial domain. Watermarking is manipulated on image pixels in the spatial domain, whereas it is performed on image frequency components in the transform domain. The human perception is further classified into invisible and visible. Reversibility was categorized into reversible and non-reversible. The procedure type is divided into non-blind, semi-blind, and blind.

#### Hiding domain

A review of watermarking techniques used in eHealth (Tele-Medicine, Tele-Radiology, Consultation, Microscopic surgery) application is presented in this survey. A brief introduction of each technique has been discussed in the following sections.

##### Spatial domain techniques

In this domain, the pixel values are directly altered to embed the watermarked media. Techniques include least significant bit (LSB), local binary pattern (LBP), histogram modification, and spread spectrum techniques. In LSB, the watermark is embedded by substituting the rightmost bits of each pixel with the watermark bits values after converting the cover image pixels value into binary format. Finally, the pixels are transformed to their original value. LSB is considered the most straightforward technique with high embedding capacity [15].

In this paper, Huma et al. [71] proposed a watermarking technique based on joint watermarking and encryption for medical images. Patient information is encrypted using the output feedback. Advanced encryption standard algorithm. The embedding process of the watermark inside medical images is executed using DWT and LSB, and the testing results show that the proposed approach is secured and robust against different attacks.

Mahboubeh and Mehrabian [123] proposed a hybrid secure watermarking algorithm based on LSB, integer wavelet transform, and chaotic sequence. Using chaotic sequence in watermark encryption also achieves a high-security level, determining the embedding location and coefficient in the host image. No embedding is done inside the ROI area in the proposed algorithm, so the image is divided into ROI and RONI. Features extracted from ROI and patients’ personal and medical information are encrypted using a chaotic sequence from IC, which is embedded in the second level band of the host image. The experimental results show that the proposed algorithm has high robustness and imperceptibility compared with other state-of-the-art approaches.

Authors in this work [108] introduced a robust zero watermarking approach for medical image security to achieve robustness against geometric attacks and considered the distinguishability of similar medical images. Firstly, features are extracted from the medical image using DRZW, based on the completed local binary pattern (CLBP) to ensure robustness against geometric attacks and distinguishability. Then, the master and ownership share operator is created to ensure distinguishability and robustness, especially against geometric attacks. The proposed algorithm was applied to 200 different medical images of five types. The experimental results show that the DRZW algorithm ensures the reliability and accuracy of authentication.

This paper [55] proposed a new medical image watermarking approach based on redundant discrete wavelet transform (RDWT) and multiple histogram modification (MHM). To enhance the embedding capacity, the authors used the MHM algorithm. Then the watermark is embedded in the RDWT coefficients, which has several sub-band that provides better embedding capacity and guarantee its visual quality. The testing results proved that the proposed scheme presents better embedding capacity and robustness.

Chauhan et al. [38] present a new secure watermarking scheme for medical images based on the spread-spectrum concept. Firstly the cover image is decomposed into its frequency sub-band then a pair of pseudo-noise corresponding for each watermark pixel is embedded into each column and row sub-band. Various kinds of attacks are applied to the transmitted image in the testing phase. The experimental results showed that the technique offers more robustness than others.

In work [163], the authors illustrate multiple watermarking methods for medical images based on spread-spectrum. The telemedicine center logo and patient’s personal and medical information are used as a watermark embedded in the host image. The pseudo-noise (PN) sequences are constructed according to each watermark bit then embedded into the host image selected DWT coefficients. It has been found from the experimental results that the approach.

Gives superior performance compared with other multiple watermarking methods.

Eze et al. [49] introduced an accurate digital watermarking algorithm and tamper detection for medical image security based on Spread Spectrum in this research paper. In the proposed method, the patient personal information is used as a watermark then embedded in the medical image sub-block. The experimental results show that the proposed scheme improved accuracy and tamper detection with low computational cost.

In [72], the author proposed a robust watermarking scheme based on graph coloring and vector quantization tested on an Android phone. Before the embedding process, the medical image is segmented into ROI and RONI. RONI is selected for watermark embedding using different processes like graph generation and graph coloring. Implementing the proposed technique on an Android device is considered an achievement of imperceptibility and low complexity.

##### Transform domain

In the frequency domain mechanisms, the host media coefficients are rebuilt after the embedding process. The methodology includes DFT, DCT, RDWT, SVD, DWT, etc. [15]. The spatial-domain techniques are simpler in computation than the frequency domain but less robust against geometric attacks. The spatial and transform domain techniques are compared for evaluating some properties. Brief research work is discussed below in Table 2.

**Table 2:** Comparison of Spatial and Transform Techniques

Parameter	Spatial Domain	Transform Domain
Complexity	Low	High
Robustness	Low	High
Capacity	High	Low
Imperceptibility	Low	High
Computational Time	Low	High

In DCT, the image is decomposed into low (FL), middle (FM), and high (FH). The majority of image energy is found in the low-frequency band. In DWT, the image was segmented as LL, HL, LH, and HH. “L” stands for low, and “H” stands for high.

Furthermore, the LL sub-band was also continuously segmented to achieve other levels based on the application’s required number. In DFT, the image was resolved into cosine and sine forms and implemented watermark embedding using either template-based hiding or direct hiding. Therefore, it provides more protection against geometric attacks.

Kahlessenane et al. [83] proposed a new roust and blind watermarking technique for medical image security based on discrete wavelet transform. In this approach, the patient’s personal information is combined with the constructed coefficients of the LL sub-bands to generate the watermark bits. With the number of experiments applied on secured images to verify the invisibility and robustness of the proposed model, the experimental results show that the algorithm offers high robustness with excellent imperceptibility against geometric attacks.

Fares et al. [52] proposed two blind watermarking techniques for medical image exchange protection. The first one is based on Schur decomposition and Discrete Cosine Transformation to achieve high robustness and imperceptibility. The second one is based on Discrete Wavelet Transform and Schur decomposition. The experimental results prove that the proposed schemes are very robust against traditional attacks with high-quality watermarked images to protect the patient’s information.

This paper [24] introduced a new watermarking method for medical image protection based on wavelet transformation and fuzzy-based Region of Interest (ROI) selection. First, the fuzzy-based algorithm is applied to the cover image to determine the sensitive points and select the ROI. Second, the wavelet transform is applied to decompose the image into sub-bands for the embedding phase. Experimental results show that the proposed algorithm is robust against various kinds of attacks.

Thanki et al. [178] proposed a blind watermarking approach for medical protection, based on Discrete Cosine Transform (DCT) and Fast Discrete Curvelet Transform (FDCuT) to get different frequency coefficients. The system is tested on various types of medical images to measure its effectiveness. Experimental results proved that the scheme is robust to different geometric attacks and others compared with the state of art schemes. Singh et al. [164] proposed a new hybrid watermarking technique in the transform domain for medical images security. To achieve high imperceptibility, robustness, and capacity. The authors combined nonsubsampled contourlet transform (NSCT) with singular value decomposition (SVD) and discrete cosine transform (DCT). The NDCT is used to increase hiding capacity and robustness against geometric attacks. The electronic patient record is embedded into the selected medical images sub-band. The proposed algorithm achieved high robustness against signal processing and geometrical attacks.

#### Human perception

The watermark is an effect embedded in an image that may be invisible or visible to identify the ownership of the given data [59]. Digital watermarking has proved to be a promising technology for enhancing the copyright protection of images. Table 3 summarizes the techniques from a human perception point of view.

**Table 3:** Watermarking Approaches

Technique	Definition	Application
Visible watermark	It is mainly a semi-transparent text or image embedded in the cover image.	Logo on TV channel
Invisible watermark	The watermark is embedded to be invisible to the user’s naked eye, but it can easily be identified that the image is watermarked or not by the computer devices.	Copyright protection applications
Robust watermarking	Robust watermarking techniques are resistant to multiple various attacks. The authors in [125] applied the SVD method to the medical image watermarking technique based on FDCuT-DCT. The proposed algorithm has good imperceptibility so that it can maintain the quality of medical images	used in copyright protection
Fragile watermarking	Watermark allows being demolished by straightforward alteration easily. Any slight change in the watermarked image will cause the watermark to break, low imperceptibility	integrity verification, and authentication applications.
Semi-fragile watermarking	Defend on the covered data against deliberate attacks but is frail against malignant attacks.	Copyright protection but with Low imperceptibility
Zero watermarking	Unlike other watermarking techniques, the watermarking process does not modify the image, as the watermark does not embed in the image itself [141].	Copyright protection, authentication, and tamper detection for medical data
Hybrid watermarking	It is a combination of robust and fragile techniques	Integrity verification, copyright protection, and authentication concurrently.

In this paper, Mata-Mendoza et al. [112] proposed a hybrid and robust watermarking technique for medical images security based on quantization index modulation algorithm and forwarding error correction to embed metadata as a robust-imperceptible watermarking. The experimental results of the proposed technique show that it’s efficient and robust against various traditional attacks with high imperceptibility.

In this paper [56], the authors proposed a fragile watermarking scheme for tamper detection in a medical image and region of interest protection. The medical image is divided into packets. The cyclic redundancy check code (CRC) based on standard polynomial generator CRC-32 is utilized to be applied on each packet to generate a watermark. Results of experiments prove the imperceptibility and robustness against aggressive attacks.

#### Hybrid techniques

Recently, researchers have proposed a hybrid of hiding and cryptography techniques. In [174], the authors proposed a multilayer watermarking scheme based on chaotic encryption and SVD, DWT, DCT by encrypting the watermarked image. In [176], the authors proposed a robust, non-blind, and high payload using FRT and SVD hybridized with Arnold scrambling to add additional security for the proposed algorithm. Balasamy et al. [23] proposed a hybrid algorithm between watermark and encryption to increase medical image security. The experimental results of this method illustrate that the proposed scheme has better authentication than others.

Alzahrani et al. [14] proposed a robust watermarking technique to protect medical image copyright in a hybrid domain. The proposed scheme is a hybrid of three algorithms, Discrete Cosine Transform (DCT), Singular Value Decomposition (SVD), and Discrete Wavelet Transform (DWT). Firstly the medical image is divided into a region of interest (ROI) and a region of non-interest (RONI), then get high and low-frequency bands from RONI using DWT, the appropriate block for embedding are selected using Human Visual System (HVS). The experimental results proved that the proposed technique has high imperceptibility as well as robustness.

Accurate polar complex exponential transform (APCET) based on Gaussian numerical integration (GNI) describes two similar medical images simultaneously. To overcome the limitation in most zero watermarking schemes, which only support protection for one image at a time. Chang et al. [110] proposed a new zero watermarking for protecting two similar medical images using TAPCET and chaotic mapping. The experimental results show that the proposed scheme is robust against various geometric and image processing attacks.

The current paper [3] uses a new medical image watermarking technique based on the blue monkey meta-heuristic and honey algorithms. The first is used to select the best hiding location in the host image. The second, utilized for watermark encryption before embedding. Furthermore, various image processing and geometric attacks are applied to host images for testing. The experimental results of the proposed algorithm demonstrate that it is robust and secure against most attacks.

Hosny et al. [67] introduce new geometrically invariant multiple zero-watermarking schemes to overcome the lack of security found in single watermarking algorithms for medical image security based on Gegenbauer moments of fractional orders (FrMGMs). FrMGMs are used to extract the geometrically invariant features from medical images and form the feature vector using the magnitude of these moments. The extracted features XORed with scrambled watermark image to construct ownership share. The proposed approach provided higher robustness to geometric and other attacks.

#### Procedure type

The main challenge of designing a watermarking technique for medical image security should be blind or non-blind. More patient information has been embedded into the cover image to achieve robustness against various attacks. Table 4 shows techniques of procedure type classifications.

**Table 4:** Watermark Procedure Techniques

P.O.C	Blind	Semi-Blind	Non-Blind
Definition	No need for original information to extract the watermark.	It only requires a secret key and a watermark to extract the data.	Both secret key and watermark and cover image are required to extract the watermark.
Applications	• Healthcare • Copyright protection • E-voting • Remote education • Image authentication • Tamper detection	• Copyright protection • Image authentication • CAD models • Content privacy • Wireless and Adhoc systems	• Copyright protection

Soualmi et al. [166] presented a blind medical image watermarking technique. Based on combining the Weber descriptors (WDs), DCT transform, and Arnold chaotic map. Firstly, The DCT coefficients of each block are extracted using DCT, and then the watermark is scrambled using Arnold’s chaotic map. The scrambled watermark embedded in DCT proper coefficients were selected based on WDs. The proposed technique improves the robustness against different attacks compared with other state-of-the-art algorithms.

Soualmi et al. [167] proposed a new watermarking scheme for medical image security based on MinEigen value features, chaotic sequence, and Quantization Index Modulation (QIM). Then embed the watermark bits in 3 × 3 blocks selected by using MinEigen value. The experimental results showed that the proposed approach is robust and imperceptible against compression attacks. In this work, [46], the authors proposed a hybrid image quality degree algorithm based on singular value decomposition (M-SVD) for both grey and color images. The proposed algorithm embeds a watermark in the medical image then applies several geometric and image processing attacks. Results show that the M-SVD algorithm has perfect results in medical images in both color and grey images.

#### Reversibility

Abbasi et al. [1] proposed a medical image watermarking system. Firstly the image is divided into two regions region of interest (ROI) and region of noninterest (RONI). The authors embed two different watermarks, one fragile watermark in ROI and the other robust watermark in RONI. For casting the watermark, simple LSB. The system was tested using different CT medical images. The simulation results prove that the proposed system provides higher security and confidentiality of medical images.

Shehab et al. [155] proposed a new fragile watermarking technique for medical image authenticity. The proposed technique can locate and recover tampered images based on SVD and LSB. SVD help in improving the medical image authentication and detecting different attacked area of the watermarked image. Different types of attacks are applied to the testing datasets. The experimental results prove that the proposed scheme improves tamper localization and original image recovery compared with other state-of-the-art methods.

## Performance metrics

**Fig. 11:**
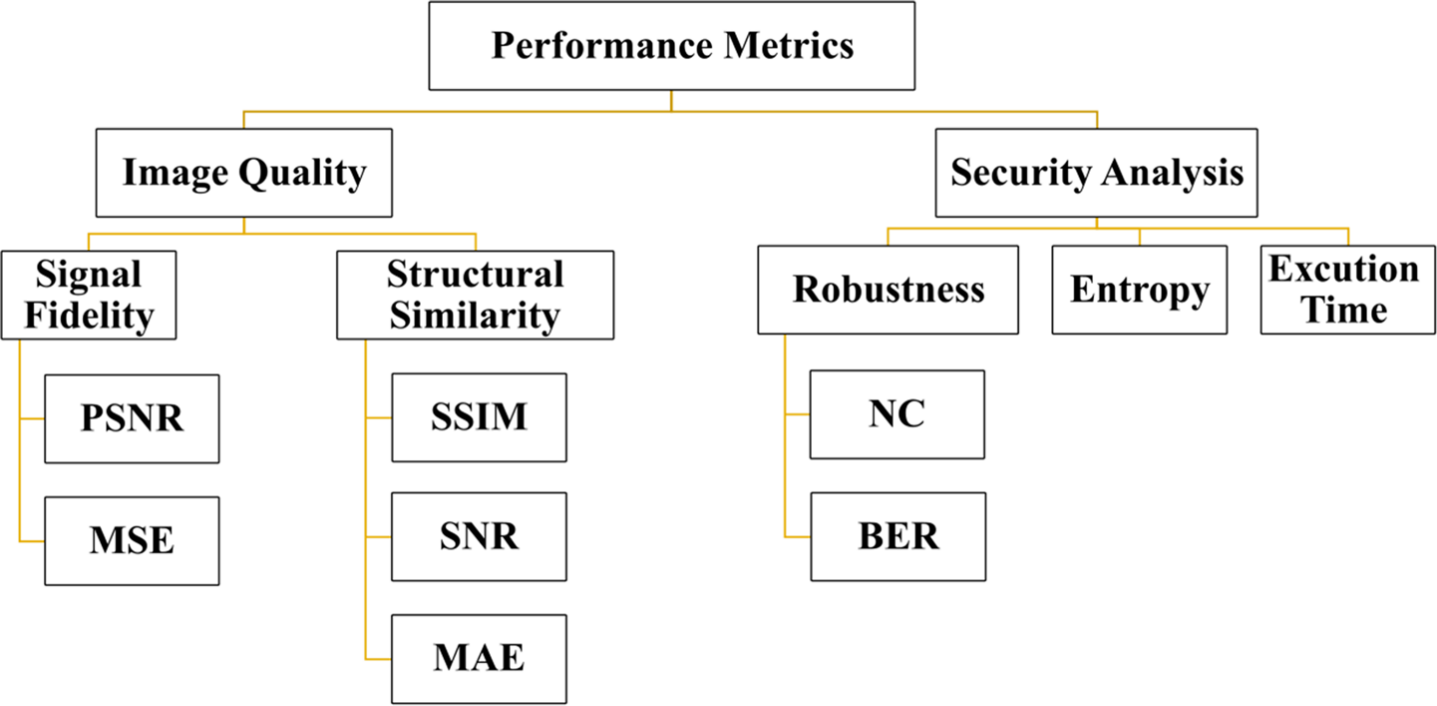
Performance Metrics

The proposed algorithms can be measured from different sides as computational speed, time complexity, quality of encrypted and decrypted images, and robustness. These metrics are summarized in Fig. 11. In the following lines, some of these metrics have been discussed in Table 5.

**Table 5:** Performance Metrics

Reference	Measures	Formula	Optimum Value
[33]	PSNR	PSNR criteria are used to know the proposed algorithm’s imperceptibility according to how the watermarked image and the original image are similar. A high PSNR value means a high similarity between the two images. It is represented as, $\mathrm{PSNR}={10}_{\mathrm{log}}\frac{(255)^2}{\mathrm{MSE}}$	High as possible
	MSE	Mean Square Error is: $\mathrm{MSE}=\frac{1}{\mathrm{X}\ \mathrm{x}\ \mathrm{Y}}{\sum}_{\mathrm{i}=1}^{\mathrm{X}}{\sum}_{\mathrm{j}=1}^{\mathrm{Y}}{\left({\mathrm{I}}_{\mathrm{i}\mathrm{j}}-{\mathrm{W}}_{\mathrm{i}\mathrm{j}}\right)}^2$	Range from 0 to 1 Ideally =0 This value means the two images are identical
	NC	NC is used in calculating the similarity between the extracted and the original watermark coefficient value range between 0 and 1. It can be mathematically represented as $\mathrm{NC}=\frac{\sum_{\mathrm{I}=1}^{\mathrm{X}}\sum_{\mathrm{J}=1}^{\mathrm{Y}}\left({\mathrm{W}}_{\mathrm{org}\mathrm{ij}}\ \mathrm{x}\ {\mathrm{W}}_{\mathrm{recij}}\right)}{\sum_{\mathrm{I}=1}^{\mathrm{X}}\sum_{\mathrm{J}=1}^{\mathrm{Y}}\left({\mathrm{W}}_{\mathrm{org}-\mathrm{i}{\mathrm{j}}^2}\ \right)}$	Ideally, NC = 1 but 0.7 is acceptable
[186]	NPCR	$\mathrm{NPCR}:\mathrm{N}\left({\mathrm{C}}^1,{\mathrm{C}}^2\right)=\sum_{\mathrm{I},\mathrm{J}}\frac{\mathrm{D}\left(\mathrm{i},\mathrm{j}\right)}{\mathrm{T}}$	Range from 0 to 100 Ideally =100
	SSIM	SSIM is one of the most recently used criteria to find similarities between the original and the watermarked image	Ranged from 0 to +1. Ideally =1
[127]	UACI	$\mathrm{UACI}:\mathrm{U}\left({\mathrm{C}}^1,{\mathrm{C}}^2\right)=\sum_{\mathrm{I},\mathrm{J}}\frac{\mid {\mathrm{C}}^1\left(\mathrm{i},\mathrm{j}\right),{\mathrm{C}}^2\left(\mathrm{i},\mathrm{j}\right)\mid }{\mathrm{F}.\mathrm{T}}$	Range from 0 to 100 Ideally =100
[159]	BER	$\mathrm{BER}=\frac{\mathrm{number}\ \mathrm{of}\ \mathrm{incorrectly}\ \mathrm{decoded}\ \mathrm{bits}}{\mathrm{Total}\ \mathrm{number}\ \mathrm{of}\ \mathrm{bits}}$	Small as possible Ideally =0

### Image quality measurement

One of the essential performance metrics that must be discussed. Some image encryption algorithms may result in some deformation in the ciphered images, which affects data reliability. Decrypted image accuracy is essential in some medical and military applications. Affecting the ROI leads to misdiagnosis. This metric, applied by evaluating the relationship between source and decrypted images, represents the quality of ciphered images. The widespread image aspect measurements are peak signal-to-noise ratio (PSNR), bit correct ratio (BCR), structural similarity index measure (SSIM), signal-to-noise ratio (SNR), mean absolute error (MAE), mean squared error (MSE), and standard dynamic range (SDR). Despite signal fidelity, metrics are often disputed because they have no regard for the image signal’s nature. They are still widely used as image quality measures [193]. The various measures utilized are discussed next.

In 2017, Pak and Huang [128] applied peak signal-to-noise ratio (PSNR) metrics to evaluate their proposed algorithm. As known, when the value of PSNR is large, this means that the tested algorithm results in high decrypted image quality. The experimental results of the utilized encipher technique have outstanding accomplishments in facing various attacks and noise. Li et al. [97] also applied this metric to their algorithm and got results similar to the previous test.

The MSE is used to evaluate the level of defense on encipher images. Ran et al. [136] used MSE criteria between the original image and ciphered image. Also, Wang et al. [188] used this metric in their grading. The retrieved images from their work are heavily deranged by noise but can still recognize the primary information inside the image affected by the noise. This experiment shows that the introduced methodology can withstand noise but within a certain level.

In 2018, X. Li et al. [98] preferred to use one of the structural similarity metrics, SSIM, to measure their success in reconstructing encrypted images. The results show that the introduced scheme gives high SSIM results, which give high image quality. The SSIM values calculated by the authors are nearest to one, and SSIM values range between 0 and 1, which means no distortion in the reconstructed frames when the value is close to 1.

Khan et al. [92] proposed a technique to improve the signal-to-noise ratio’s strength (SNR). They prepared new statistical criteria, mean absolute error (MAE), and, as the encryption security increased with a more considerable MAE value, the authors’ SNR result proved the proposed algorithm’s efficiency.

### Metrics formula and optimum value

## Data sets

Digital Imaging and Communications in Medicine (DICOM) was considered as criterion formate for medical images. DICOM standard is used to guarantee a proportionate format for transmitting, processing, storing medical images. This formate facilitates sharing medical images through the internet and several servers between patients, medical centers, and vice versa [40]. Medical devices from different suppliers can transmit medical images through the network. In the following lines, many researchers’ medical images datasets in their experiments have been mentioned.

Falgun et al. [173] tested the proposed algorithm on a dataset chosen from the digital imaging and communication in medicine (DICOM) as CT, X-ray of size 1024 × 1024. In this contribution [48], the authors used large numbers of large-sized X-ray images of Pneumonia infected and healthy patients from the Pneumonia Chest X-ray of the Kaggle Competition.

Singh et al. [161] experiments were done on 565 × 584 pixels fundus and retinal images of the DRIVE free public dataset. Guzin et Arda [181], in their experiments, depend on the dataset that was gathered from the Internet and created by their research group, which contains several tampered (rotating, scaling, blurring) Grey-level medical images.

## Discussion

### Statistical analysis

**Fig. 12:**
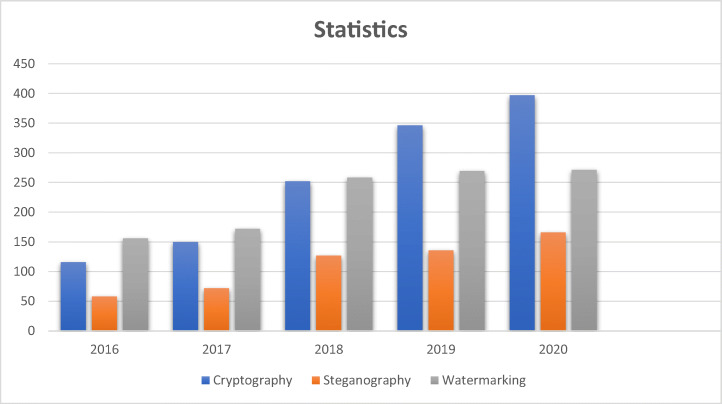
The number of published studies in the last five years

In this section, Fig. 12 shows a statistical representation of related research papers published in archived journals (Springer, IEEE Explorer, and Science Direct) of the last five years in the field of medical data security. It is concluded that medical image security is growing and has become a big challenge because of its importance.

### Medical data security requirements

Many recent efficient security methodologies have been proposed for medical data security. However, there is some limitation in its practicality. Based on previous studies, we focus on some potential challenges.

#### Cryptography

- According to the application, encryption and decryption operations applied on medical images may lead to image distortion, so the proposed technique should be lossless.
- Computational speed is considered the main challenge in encryption and decryption.
- Compression techniques can be used to compensate for the shortage in payload capacity.

#### Steganography

- Maximize embeddable regions to suit more information.
- Maintain the imperceptibility for the stego image.
- Embedding bits selection of the cover image.
- Achieve lower computational time with high robustness.
- Design steganographic systems for real-time applications with high privacy.

#### Watermarking

- Put all types of image attacks into consideration while improving or developing a watermarking technique.
- Both robustness and computational complexity of the proposed system are essential.
- ROI must be observed while embedding the watermark to not affect the features in medical images.
- The reversibility of the watermarking approach is essential in medical images.
- Hybridization between watermarking and encryption can be used to improve performance.
- Some compression techniques have been used to increase embedding capacity.

In some applications, preserving image quality is a must because it may lead to medical images diagnosis or military data transmission. Simple distortion in the image is acceptable by taking a watermarking approach, for example, selecting a watermarking technique. Fragile, blind, robust, and zero-watermarking would be used according to the application. Most sensitive image applications use zero-watermarking for security because zero-watermarking was considered one of the approaches that don’t cause image distortion ratio. The watermark does not embed inside the host image, as most traditional watermarking techniques do.

The primary step for zero-watermarking is how to extract special and robust image features, robust against various image attacks, to reconstruct the watermark and verify copyright. In [80, 144], the authors discussed various global and local features. In global features, the processing is performed on whole host image pixels, as Geometric Moment Features, Space-Time Volumes (STV). In local features, the processing is performed on each pixel as GLCM, SIFT, SURF [142], and Local binary Pattern and the ability of each technique to resist attacks. Hosny et al. [69] proposed highly accurate, fast, and robust techniques for gray and color images based on computing the moments of (PCET) for the host image. The results proved that the proposed algorithm gives higher accuracy for watermarked image reconstruction with high visual imperceptibility and robustness to common attacks. Hosny et al. [68] proposed a robust watermarking algorithm based on new fractional-order exponent moments applied on color images.

### Performance analysis and discussion

As shown above, various approaches are proposed for medical images and ordinary images with copyright protection and tamper detection as cryptography, steganography, and watermarking. Nothing proved that any of these techniques is the best for image security, so we can say that deciding to use any one of these approaches is based on application type and data sensitivity Table 6, Table 7, and Table 8 presents the most current state of art medical image security approaches.

**Table 6:** Performance discussion for cryptography-based medical image security techniques

Ref	Proposed method	Method	Time Complexity	Performance							Pros
				UACI	NPCR	NPCR	SNR	PSNR	MSE	SSIM	
[134]	Multiscale transform-based secured joint efficient medical image compression-encryption using symmetric key cryptography and ebcot encoding technique	Multiscale transform using ebcot encoding technique and symmetric key cryptography	Low Computational Complexity	NA	NA	NA	76.869	NA	0.0009	0.9999	Compression time is not affected by encryption time.
[130]	A novel visual medical image encryption for the secure transmission of authenticated watermarked medical images	Medical image is encrypted visually with the fingerprint using IWT.	T = 37^n^ Robust to brute force attack	0.000014		40.7344	NA	30 dB	NA	NA	The proposed method presents high integrity with high imperceptibility for increasing security and authentication using fingerprint images.
[30]	Secure visual cryptography for medical image	This paper fuse the concept of modified cuckoo search	Has low computational complexity	NA	NA	NA	NA	49.51	0.0171	NA	The proposed scheme provides Reversibility and robustness.
[63]	A privacy-preserving cryptosystem for IoT E-healthcare	Chaotic Map (PWLCM) and Logistic Map	T = 0.95 Seconds For akeyframe [640x480x3] With encryption speed =970 KB/Ssec	33.465	NA	99.609	NA	73.5965	0.0028	NA	The proposed algorithms effectively resist various attacks besides keeping the confidentiality of the medical in-Formation.
[34]	Medical image encryption using edge maps	Encryption algorithm using edge map	Time various from 0.0129 to 1.846 s for images 8 × 8 to 512 × 512	0.3348	0.9960	NA	NA	NA	NA	NA	The proposed algorithm has a considerable defense against the Bruce-force attack with higher pixel correlation, stronger key sensitivity, and error robustness
[95]	Medical image encryption based on improved ElGamal encryption technique	ElGamal encryption scheme is designed to encrypt the medical image	Time various from 0.093750 s to 1.718750 s for images 256 × 256 to 1024 × 1024	33.41	NA	99.62	NA	NA	NA	NA	It has been proved that the proposed model presents good characteristics of a strong cipher with sound encryption and decryption speed.

**Table 7:** Performance discussion for Steganography-based medical image security techniques

Ref	Performance						Cons
	Complexity	Imperceptibility	Visual Quality		Embedding Capacity	Steganalysis Resistivity	
			PSNR	MSE			
Santhi and Dheepta [147]	High	Imperceptible	71.3896	0.0048	Approx. 0.8 bpp	Preserve the Histogram, Chi-Square	The method needs more recourses to be applied and due to high computational cost and low payload capacity.
Martiri et al. [111]	Low	Not Imperceptible	NA	NA	1 bpp	RS analysis	The proposed algorithm is not robust to image processing attacks.
Al-Dmour and Al-Ani [9]	Low	Imperceptible	56.81	0.136	1 bpp	Histogram, STC	The authors did not care about the ROI region in this method, which is very important when dealing with medical images.
Jain et al. [74]	High	Imperceptible	86.55	0.0005	3.6 bpp Raised 43%	RS analysis	The suggested method gives good results for a small amount of data.
Jain and Kumar. [75]	High	Imperceptible	79.36	0.0021	1.03 bpp Percentage 36%	NA	The algorithm is not appropriate for real-time applications due to the high complexity
Naidu et al. [122]	Low	Imperceptible	73.02	0.0049	Approx 1.55 bpp	Pixel Difference Histogram	The drawback of the proposed scheme was that it did not separate the image into ROI and RONI Regions.
(Nguyen et al., 2015) [124]	High	Imperceptible	Approx 46	NA	1.5 bpp	SPAM with Ensemble Out of Bag (OOB)	NA
(S. Shen et al., 2015) [156]	Low	Not Imperceptible	36	0.045	1.5 bpp	RS analysis, Pixel Difference Histogram	Not Imperceptible
(Grajeda- Marín et al., 2016) [57]	High	Imperceptible	38.33	NA	2.14 bpp	NA	NA

**Table 8:** Performance discussion for watermarking-based medical image security technique

Reference	Evaluation Metric	Geometric Attacks				Compression		Image Processing Attacks								
		Rotation	Translation	Cropping	Scaling	JPEG	JPEG2000	Gaussian noise	Salt & Pepper noise	Median filter	Gaussian filter	Average Filter	Contrast	Sharpening	Blurring	Hist. Equalization
[79]	BER	NA	NA	NA	NA	NA	NA	NA	NA	NA	NA	NA	NA	NA	NA	NA
	PSNR	NA	NA	0.989	>40	>40	>40	NA	>40	>40	>40	NA	NA	NA	NA	NA
	NC	NA	NA	0.73814	0.99744	0.99853	0.99629	NA	0.99280	0.99115	0.95951	NA	NA	NA	NA	NA
	SSIM	NA	NA	41.3784	Inf	Inf	inf	NA	Inf	Inf	inf	NA	NA	NA	NA	NA
[90]	BCR	NA	NA	NA	NA	NA	NA	NA	NA	NA	NA	NA	NA	NA	NA	NA
	BER	NA	NA	NA	NA	NA	NA							NA	NA	NA
	PSNR	NA	NA	NA	NA	NA	NA	NA	24.3557	NA	NA	NA	NA	NA	NA	NA
	NC	NA	NA	NA	NA	NA	NA	NA	NA	NA	NA	NA	NA	NA	NA	NA
	SSIM	NA	NA	NA	NA	NA	NA	NA	NA	NA	NA	NA	NA	NA	NA	NA
[160]	BER	NA	NA	NA	NA	NA	NA	NA	NA	NA	NA	NA	NA	NA	NA	NA
	PSNR	NA	NA	NA	NA	NA	NA	NA	34.88			NA	NA	NA	NA	NA
	NC	0.8269		0.9859	0.9906	0.9882	NA	NA	0.9579	0.979	0.8184	NA	0.9246	NA	NA	NA
	SSIM	NA	NA	NA	NA	NA	NA	NA	NA	NA	NA	NA	NA	NA	NA	NA
[11]	BER	NA	NA	NA	NA	NA	NA	NA	NA	NA	NA	NA	NA	NA	NA	NA
	WSNR	0.0982		8.1109	NA	NA	NA	27.0916	32.1470		0.2802			0.2801		
	PSNR	5.9136	NA	13.7111	NA	NA	NA	19.9203	24.6345	NA	5.9212	NA	NA	5.9214	NA	NA
	NC	NA	NA	NA	NA	NA	NA	NA	NA	NA		NA	NA		NA	NA
	MSSIM	0.2728	NA	0.7391	NA	NA	NA	0.8212	0.9304	NA	0.1030	NA	NA	0.1031	NA	NA
[158]	BER	NA	NA	NA	0.444	0.96		0.55	0.48	0.93	NA	NA	NA	NA	NA	0.14
	BCR	NA	NA	NA	NA	NA	NA	NA	NA	NA	NA	NA	NA	NA	NA	NA
	PSNR	NA	NA	NA	NA	NA	NA	NA	NA	NA	NA	NA	NA	NA	NA	NA
	NC	NA	NA	NA	0.7375	0.9905	NA	0.7267	0.6069	0.9985	NA	NA	NA	NA	NA	0.569
	SSIM	NA	NA	NA	NA	NA	NA	NA	NA	NA	NA	NA	NA	NA	NA	NA
[175]	BER	NA	NA	NA	NA	NA	NA	NA	NA	NA	NA	NA	NA	NA	NA	NA
	PSNR	NA	NA	NA	NA	NA	NA	NA	NA	NA	NA	NA	NA	NA	NA	NA
	NC	0.9986	NA	NA	0.9986	0.9986		0.9539	0.9528	0.9826	0.9989	NA	NA	NA	NA	0.9980
	SSIM	NA	NA	NA	NA	NA	NA	NA	NA	NA	NA	NA	NA	NA	NA	NA
[26]	BER	NA	NA	NA	NA	NA	NA	NA	NA	NA	NA	NA	NA	NA	NA	NA
	PSNR	29.9297	NA	35.9698	31.0447	29.2960	NA	NA	30.04444	30.0422	41.3355	4,908,827	NA	28.6619	464,742	18.2224
	NC	NA	NA	NA	NA	NA	NA	NA	NA	NA	NA	NA	NA	NA	NA	NA
	SSIM	0.981	NA	0.999	0.955	0.978	NA	NA	0.935	0.974	0.964	0.991	NA	0.959	0.956	0.847

## Conclusion

A brief discussion on medical data types. Medical image security plays a significant role in various eHealth applications, including storage, retrieval, identity theft, and data management. In this paper, we have introduced the latest development of medical image security algorithms in recent five years and analyzed the challenges of medical images security. Some famous image attacks have been listed, which are applied to data to test the proposed algorithms. Some medical image security approaches as cryptography which is presented in spatial and frequency domains, a detailed classification of steganography are presented, and several steganography approaches have been discussed, also a comprehensive survey for watermarking types, characteristics, and medical image watermarking approaches. We have discussed these approaches and their characteristics, kinds, requirements, working mechanisms for each approach, and their potential issues to support further research in this area. Also, some medical images datasets utilized by many researchers have been mentioned.

Furthermore, some performance metrics for algorithm evaluation have been demonstrated. With the help of our survey, researchers may propose new security methodologies to secure the transmission of medical data to service eHealth applications (Tables 9 and 10).

**Table 9:** Average of evaluation metrics for various watermarking-based medical image security techniques

Proposed Method	Method	Performance	Drawback
Jia and Zhou [79]	LSB, SHA-265, MAC, AES,	Blind, fragile	High computational complexity
Kelkar and Mehta [90]	LBPTI and RDH	Reversible and Secure	Low embedding capacity
Singh and Kumar [160]	SVD, DWT, BPNN	Imperceptible, robust, and secure	Computational complexity is high
Al-qdah [11]	SVD, DWT	Blind, secure	Watermark, not robust to some attacks.
Singh, Dave [158]	DCT, DWT,	PSNR = 35.84 dB, NC = 0.9992	Computational complexity is high.
Thakur, Singh [175]	RDWT, NSCT, chaotic encryption	Robust and Secure	Embedding capacity can be enhanced.
Bamal and Kasana [26]	FWT, SLT, ANN, AES,		Low Imperceptibility

**Table 10:** Abbreviations

Acronyms	
PSNR	Peak Signal to Noise Ratio
SSIM	Structural Similarity Index
BER	Bit Error Rate
NC	Normalized Coefficient
NPCR	Number of Pixels Changes Rate
UACI	Unified Average Change Intensity
MRI	Magnetic Resonance Imaging
CT	Computed Tomography
US	Ultrasonography
ROI	Region of Interest
RONI	Region of Non-Interest
CFI	Choquet Fuzzy Integral
EA	Evolutionary Algorithms
ACM	Arnold Cat Map
ICA	Independent Component Analysis
GA	Genetic Algorithm
GT	Gyrator Transform
DCT	Discrete Cosine Transform
FrFT	Fractional Fourier Transform
MAE	Mean Absolute Error
SDR	Standard Dynamic Range
SNR	Signal to Noise Ratio
PVD	Pixel Value Differencing
LSB	Least Significant Bit
DWT	Discrete Wavelet Transforms
HVS	Human Visual System
IWT	Integer Wavelet Transform-Based Steganography
AI	Artificial Intelligence
ML	Machine Learning
NN	Neural Networks
SVM	Support Vector Machine
LBP	Local Binary Pattern
HUGO	Highly Undetectable steGO
SSIM	Structural Similarity Index Measure
POV	Pair of Value
WS	Weighted Stego Steganalyser
MLSB-WS	Multi Bitplane Weighted Stego Steganalyser
